# Protective mental health factors in children of parents with alcohol and drug use disorders: A systematic review

**DOI:** 10.1371/journal.pone.0179140

**Published:** 2017-06-13

**Authors:** Olga Wlodarczyk, Mirjam Schwarze, Hans-Jürgen Rumpf, Franka Metzner, Silke Pawils

**Affiliations:** 1 Institute and Outpatients Clinic of Medical Psychology, Centre for Psychosocial Medicine, University Medical Centre Hamburg-Eppendorf, Hamburg, Germany; 2 Department of Psychiatry and Psychotherapy, University of Lübeck, Lübeck, Germany; Universita Cattolica del Sacro Cuore Sede di Roma, ITALY

## Abstract

Children of parents with drug and alcohol use disorders often grow up under severe stress and are at greater risk of developing psychological and social problems. However, a substantial proportion of affected children adapt to their distressing life conditions and show positive development in terms of their mental health. These children are described as resilient. One difference between resilient and maladapted children is the presence of protective factors. The aim of this systematic review is to provide an overview of the current state of the research concerning protective mental health factors in children of parents with alcohol or drug use disorders (COPAD). For that purpose, the PsychInfo, PubMed, CINAHL and ISI Web of Science databases were searched through January 2017. All the identified publications were screened using previously developed inclusion criteria. The search yielded 3,402 articles. Eleven of these publications (2003–2013) met the criteria for inclusion in the present review. Information on the studies was extracted using an extraction form. A narrative analysis was performed, and the methodological quality was examined using a checklist based on the Mixed Methods Appraisal Tool. The research identified familial, parental, child-related and biological factors that influenced mental health outcomes in affected children (N = 1,376, age range = 1–20 years). Overall, protective mental health factors are understudied in this target group. Most of the included studies were conducted in the United States and employed a cross-sectional design. A comparison of the included cross-sectional and longitudinal studies indicated consistent results related to a secure parent-child attachment. Based on the current state of the research, no causal conclusions with regard to the effectiveness of protective factors can be drawn. To develop effective prevention programs, further longitudinal studies and studies assessing the interactions between risk and protective factors are needed.

## Introduction

Epidemiological data on the number of children who are affected by drug-using or alcohol-abusing parents are largely unavailable. The number of minor children who live in families in which at least one parent has alcohol- or drug-related problems is mostly based on estimates and is highly dependent upon the applied definition of problematic alcohol or drug use. In Europe, the proportion of children under the age of 20 years with alcohol-abusing parents ranges from 5.7% in Denmark to 23% in Poland, whereas the proportion of children with drug-using parents ranges from 0.2% in Denmark and Germany to 2.4% in the United Kingdom [1]. In the United States, data from the National Survey on Drug Use and Health (NSDUH) indicate that 11.9% of children under the age of 18 years live with at least one parent with alcohol or drug use disorders [2].

These children of parents with alcohol or drug use disorders (COPAD) face a higher risk of drug involvement as well as mental health and behavioral problems [3, 4]. They showed an increased magnitude of alcohol or drug use disorders themselves compared to unaffected controls in family and longitudinal studies [5, 6]. As a direct causal link between parental alcohol and drug use disorders and child outcomes could not be identified, factors that co-occur with these disorders were examined. These factors included parenting deficits, child maltreatment, family conflict and less secure attachment patterns [4, 7]. McKeganey and colleagues [8] showed that parental substance abuse might have a negative effect on family functioning, as it impacts parenting practices and childcare. Based on interviews with 30 parents who recovered from heroin addiction, their minor children were faced with material neglect as well as drug use and dealing and were at risk of violence and physical abuse, criminal behavior and family dissolution [8]. Non-sexual child maltreatment, on the other hand, was identified as an important risk factor for mental disorders, drug use, suicide attempts, sexually transmitted infections, and risky sexual behavior in a systematic review of 124 studies [9]. Furthermore, these co-occurring factors of parental drug and alcohol use disorders increase the risk of becoming involved with child welfare services and out-of-home childcare [10, 11].

However, it is important to recognize that not all children who are exposed to significant adversity subsequently develop mental health or behavioral disorders. These children are referred to as resilient [12, 13]. Instead of a congenital trait that promises invulnerability, resilience is described as a “dynamic process encompassing positive adaptation within the context of significant adversity” [14]. Previous research identified numerous personal and social resources that contribute to resilience such as self-efficacy, positive family climate and social support [15]. These resources are also referred to as protective factors [16]. Park and Schepp [17] identified 16 protective factors in children of parents with alcohol-related disorders in a systematic review. Protective factors at the individual level of the child included older age, high self-esteem, high self-regulation, high academic and cognitive abilities, and a flexible and optimistic temperament [17]. At the parental level, a secure attachment, a positive parent-child relationship, positive and consistent parenting, and less parentification enhanced the child’s resilience [17]. Furthermore, high family cohesion, adaptability and interaction and trustworthy family members as well as social support, extra-curricular activities and later positive interpersonal relationships were identified as protective factors [17].

Despite conclusive evidence of the risks of developmental impairments, treatment concepts are still largely focused on the people with addiction themselves, whereas equally affected children have more difficulty in finding systematic attention within these treatment concepts [18]. Research on resilience in children who face parental drug and/or alcohol abuse is important, as it can inform intervention programs that are designed to mitigate the negative effects. Therefore, a systematic review of the protective mental health factors in children of COPAD is missing. The present review aims to present an overview of the current state of the research on protective factors for COPAD. In addition to alcohol-related disorders, this review includes studies that focused on parents who use illicit drugs.

## Materials and methods

### Search strategy

This work was developed following the Preferred Reporting Items for Systematic Reviews and Meta-Analyses (PRISMA) statement [19]. To promote reproducibility, all the steps were documented in a review protocol throughout the entire working process, which can be accessed in the supporting information files. Case-control, cross-sectional and cohort studies focusing on protective mental health factors in COPAD were systematically reviewed. Only studies that were published in English or German were considered for inclusion. The systematic literature search of the peer-reviewed publications was conducted in four electronic databases (PsychInfo, PubMed, ISI Web of Science, CINHAL). To ensure that the results were relevant, the search in all the databases was limited to articles that were published between January 2000 and January 2017 using the keywords that are listed below.

### Search terms

- *Protective Factor* (protective factor* OR resilience* OR coping) AND
- *Children* (child* OR infant* OR offspring* OR adolescent* OR son* OR daughter*) AND
- *Parents* (parent* OR mother* OR father* OR maternal OR paternal) AND
- *Substance* (substance* OR drug* OR alcohol* OR opioid* OR amphetamine* OR cannabis* OR sedative* OR tranquilizer* OR cocaine* OR hallucinogen* OR heroin* OR hypnotic* OR marijuana OR psychedelic* OR phencyclidine OR narcotic OR illicit drug* abuse* OR misuse OR dependence OR substance disorder OR addiction)

### Study selection process

First, the titles and the abstracts of all the articles that were identified through the database search were screened by the first (100%) and second (10%) reviewer, who selected studies dealing with the children of parents with addiction in which children were the center of the investigation. In the second step, the titles and abstracts of the pre-selected studies were rated again by both the reviewers (100%) using previously defined inclusion criteria (Table 1). If the studies seemed to be eligible for inclusion, their full text was obtained and carefully examined by both of the reviewers (100%), who applied the inclusion criteria. Studies were included when all the inclusion criteria were met (“yes”) or when information was missing or inconclusive (“unclear”). Studies were excluded when at least one of the criteria was not met (“no”). If the reviewers disagreed regarding inclusion, they discussed their opinions and, if necessary, a third reviewer became involved.

**Table 1:** Inclusion criteria (IC).

Preselection		
IC 0	(1) The study focuses on children of parents with addiction and (2) the children, not the parents, are central.	
Outcome: Association between at least one protective factor and the child’s mental health		
IC 1	Protective factor: at least one factor is examined that protects or strengthens …	
IC 2	… the child’s mental health (mental and social functioning)	
Population: children of families in which at least one parent has an alcohol or drug use disorder (NOT: especially strained samples who experienced, for example, war, flight, physical impairment/disease, mental disability, child abuse, drug use during pregnancy)		
IC 3	Children: all children and adolescents with a mean age of ≤ 21 years who are in contact with…	
IC 4	Parents: at least one parent who currently or previously had substance-related abuse or dependence involving legal and/or illicit drugs (NOT: tobacco addiction/behavioral addictions, e.g., pathological gambling)	
IC 5	The study population of the control group (case-control studies) or the initial population (cohort studies) is made up of children with lower levels of mental health and…	
IC 6	Children with lower levels of the examined protective factor.	
Publication		
IC 7	The study is original, empirical research that was published in a peer-reviewed journal (NOT: a dissertation, congress contribution)	
Study design		
IC 8	The design of the study is one of the following:	
	(retrospective or prospective) cohort study OR	
	case-control study OR	
	cross-sectional study	
	NOT: intervention study	
Diagnostics		
IC 9		Parents: formal diagnostic evaluation for a substance abuse disorder or addiction was performed in accordance with the DSM-III, DSM-IV, DSM-IV-TR, ICD-9, ICD-10, RDC OR via an instrument that allows for a valid and reliable diagnosis OR via the application of a urine test OR based on the long-term use of a substance with high addictiveness (e.g., heroin, methamphetamine) OR the sample was recruited from larger longitudinal or interventional studies evaluating the effectiveness of addiction treatments (NOT: parental diagnosis based only on the children’s perspective)
IC 10		Children: the assessment of the child’s mental health was performed via a standardized instrument that allows for a valid and reliable assessment

### Data extraction and assessment of methodological quality

Citation details (authors’ names, title, place and date of publication), information on the study design, language, recruitment strategy, sample size, dropout rate, study population (age, gender of parents and children, familial socioeconomic status, parental relationship status), parents’ and child’s mental health, applied measurements and classification systems, protective factors, and results showing associations between protective factors and the child’s mental health were extracted from the included studies using an extraction form. The methodological quality of the studies was evaluated using a checklist (Table 2) based on the Mixed Methods Appraisal Tool (MMAT) [20]. The MMAT is a reliable and valid instrument that assesses the methodological quality of studies with various designs [21]. The methodological quality of the included studies was assessed by two reviewers (100%) according to the global assessment of methodological quality by Hölzel and colleagues [22]. As all the included studies already met the IC8 quality criteria, which is a valid and reliable measurement of parental drug or alcohol abuse disorders, the general methodological quality of all the included studies was already rated as ‘low quality’ at a minimum. Cross-sectional studies that met one additional criterion (of three) were rated ‘medium quality’. Cross-sectional studies that met two or more criteria were rated “high quality”. Cohort studies or case-control-studies that met three (of four) or more additional quality criteria were rated “high quality”, whereas studies that met two additional criteria were rated “medium quality”. When only one additional criterion was met, the studies were rated “low quality”.

**Table 2:** Assessment of the methodological quality of the included studies.

External validity		YES	NO	UNCLEAR	NOT APPLICABLE
1.	Are the study participants representative of the population under examination?	(+)	(-)	(0)	(/)
Measurement bias					
	Were valid and reliable measurement used to assess:				
2.	…the protective factors?	(+)	(-)	(0)	(/)
3.	…parental drug abuse disorder or addiction?	(+)	(-)	(0)	(/)
Attrition bias (cohort studies)					
4.	Was the dropout rate in both groups acceptable (≤ 20%)?	(+)	(-)	(0)	(/)
Selection bias (case-control studies)					
5.	Were the participants in the groups comparable with respect to possible confounders?	(+)	(-)	(0)	(/)

### Synthesis of the results

A narrative analysis was performed to synthesize the data that were extracted from the included studies. An association between a protective factor and the child’s mental health was considered to be statistically significant when p < 0.05.

## Results

**Fig 1:**
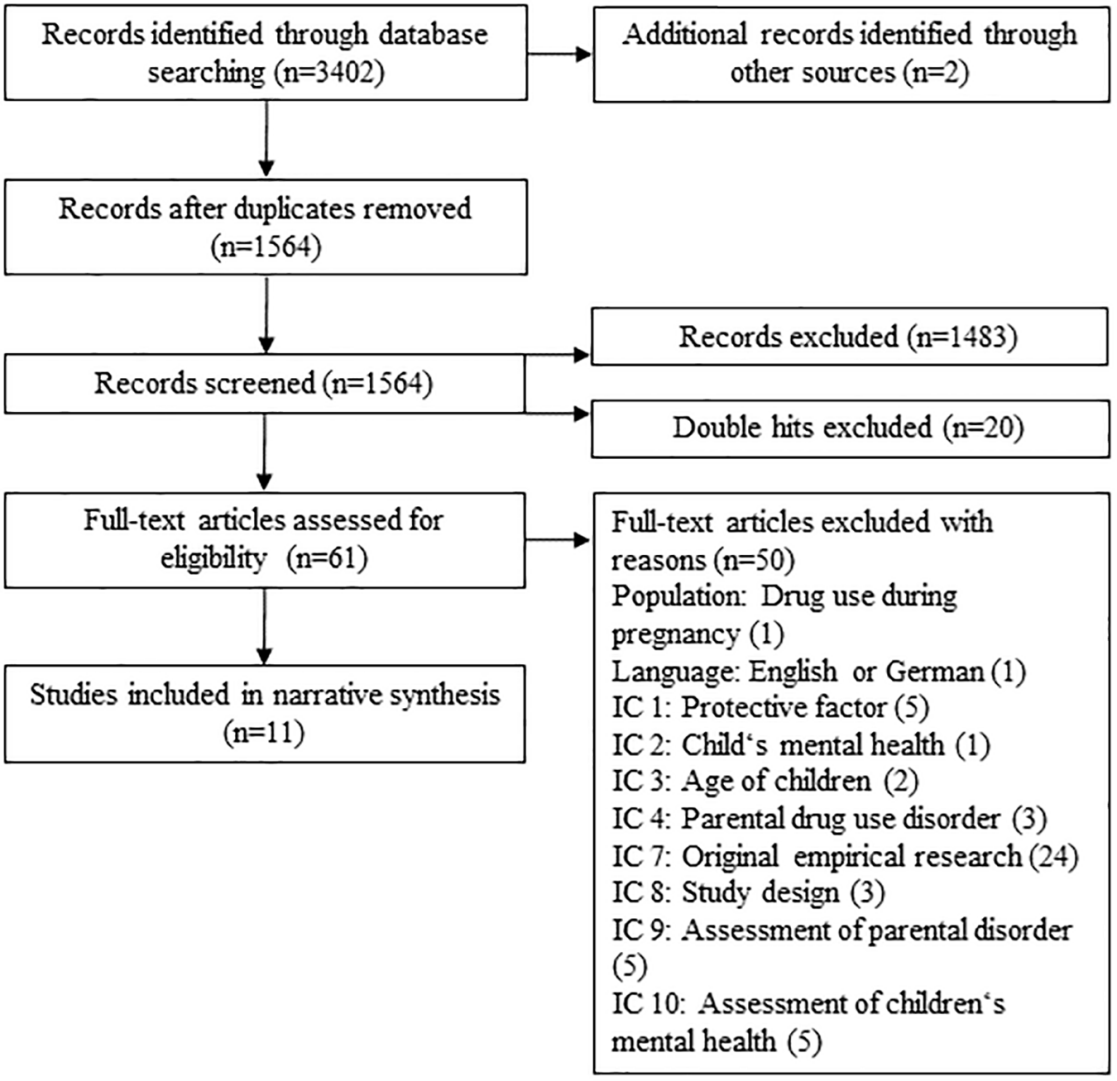
Flow of information through the different phases of the systematic review.

The database search identified 3402 potentially relevant publications. After the duplicates were removed, 1564 publications were included in the screening process. After manual screening, 1483 publications were excluded using previously defined inclusion criteria. Additionally, 20 publications were excluded, as they were double publications. Sixty-one publications were included in the full-text analysis. Non-relevant studies were excluded due to their not meeting the inclusion criteria. After the full-text analysis, 11 publications that analyzed 13 protective factors were included in the narrative synthesis of the systematic review. An overview of the selection process, including the reasons for the exclusion of studies as part of the full-text analysis, is provided in Fig 1.

### Overview

The included manuscripts were published between 2003 and 2013. All the studies were conducted in the United States, with one exception, which was performed in Israel [23]. Six cross-sectional studies [23–29], one case-control study [30], and four cohort studies [31–33] were included in the analysis. The sample size of the children ranged from *N* = 22 to *N* = 365. Overall, *N* = 1,376 children and adolescents were assessed in the 11 studies. The proportion of female children ranged from 36% to 60%. The mean age of the children ranged from 1.0 to 20.2 years. An overview of the characteristics of the selected studies is presented in Table 3. Of the 11 included studies, six assessed parental alcohol abuse or addiction [26, 29–33]. Five studies investigated disorders involving the illicit drug use of heroin, cocaine, any kind of injected drug, and methamphetamine [23–25, 27, 28]. The effects of paternal addiction only were examined in three studies [24, 31, 33]. One study was limited to the effects of maternal addiction [25]. The remaining studies lacked information on the gender of the drug-using parents. Externalizing mental health problems (e.g., antisocial and aggressive behavior) and internalizing mental health problems (anxiety and depression) were the primary mental health outcomes of the children in eight of the included studies. In all the other included studies, the primary outcomes of children’s mental health were consumption or abuse of alcohol and other drugs. In most of the studies, standardized questionnaires were used to assess the children’s mental health. In four of the included studies, the children’s mental health outcomes were assessed by the children. In three studies, it was evaluated by the children and the parents. In two studies, it was assessed by the parents. In one study, it was evaluated by the parents and teachers, whereas it was assessed by teachers only in another study.

**Table 3:** Overview of the studies that were included in the systematic review.

First author (year)	Recruitment	Study design	Parents N (% female)/ Age (SD)	Children N (% female)/ Age (SD)	Diagnostic instrument for parental diagnoses	Diagnostic instrument to assess children’s mental health outcome
Brook (2003)	Father-child dyads; outpatient substance abuse/AIDS treatment programs & general community	Cross-sectional study	204 (0)/42 (5.8)	204 (52)/16 (2.7)	Injected drug use, drug and alcohol abuse; Structured interview consisting of widely used scales	Alcohol use; Structured interview consisting of widely used and valid scales (respondent: child)
Conners-Burrow (2013)	Mother-child dyads; intake assessment, substance abuse treatment program	Cross-sectional study	79 (100)/NOS	79 (42)/4.0 (1.3)	Substance abuse (illicit drugs, alcohol); Addiction Severity Index [34]	Emotional and behavioral problems; Devereux Early Childhood Assessment [35] (respondent: teacher)
Edwards (2006)	Father-child dyads; ongoing longitudinal study	Cohort study (2 years)	91 (0)/33.0 (5.9)	117 (52)/ (T1) 12 months, (T2) 24 month, (T3) 36 month	Alcohol abuse/ dependence; Self-report instrument based on the UM-CIDI [36, 37]	Externalizing, internalizing, behavior problems; Child Behavior Checklist [38] (respondent: parent)
El-Sheikh (2003)	Families; advertisements, community sample	Cross-sectional study	NOS	75 (55)/9.4 (2.1), Controls: 141 (46)/9.5 (1.9)	Problematic alcohol consumption; Michigan Alcoholism Screening Test [39]	Externalizing, internalizing, social problems; Cognitive, academic functioning; Personality Inventory for Children [40] (respondent: parent, teacher)
Heitzeg (2008)	Families; ongoing prospective community study	Case-control study	NOS	11 (36)/17.5 (1.3), Controls: 11 (55)/18.4 (1.0)	Alcohol use disorders; DSM-IV	Externalizing behavioral problems; Alcohol use disorder risk; Youth Self-Report [41], Drinking and Drug History Form for Children [42] (respondent: child)
King (2004)	Families; court records, telephone screening, medical insurance questionnaire, ongoing longitudinal study	Cohort study (approx. 8 years)	NOS	365 (47)/ T1: 12.7 (1.45), T3: 15 (NOS), T4: 20.3 (NOS)	Alcohol abuse or dependence; Diagnostic Interview Schedule Version III [43], Family History Research Diagnostic Criteria [44]	Drug abuse or dependence other than alcohol or tobacco; Diagnostic Inventory for Children [45], Diagnostic Interview Schedule Version III [43] (respondent: child, parent)
McCauley Ohannessian (2010)	Father-child dyads; ongoing longitudinal study	Cohort study (10 years)	240 (0)/NOS	240 (60)/16.7 (1.36)	Alcohol dependence; Semi-Structured Assessment for the Genetics of Alcoholism [46]	Alcohol abuse; Michigan Alcoholism Screening Test [47] (respondent: child)
Peleg-Oren (2008)	Father-child dyads; outpatient rehabilitation centers	Cross-sectional study	72 (0), Age range: 21–40 (71), 41+ (29)	72 (47)/ Age range: 8–9 (35%), 10–11 (65%)	Substance use and dependence; DSM-IV	Psychological distress: fears, anxiety, sadness, loneliness, depression; Emotional Distress Scale (developed by Peleg-Oren & Rahav); Adjustment: psychological, social and everyday home functioning; Adjustment of the Child in the Family Scale [48] (respondent: child, parent)
Pilowsky (2004)	Families; AIDS clinic, support groups for HIV-positive individuals	Cross-sectional study	25 (68)/37 (6.9), Controls: 66 (62)/37.5 (5.9)	NOS (69)/8.2 (1.6), Controls: NOS (47)/8.6 (1.8)	Injected drug users; Drug users with a history of injection	Child Behavior Checklist [41]; Schedule for Affective Disorders and Schizophrenia for School-Age Children [49] (respondent: child, parent)
Sheridan (2011)	Families; families involved with Children and Family Services	Cross-sectional study	NOS	41 (44)/ 10 (NOS)	Methamphetamine addiction; NOS	Anxiety, depression, anger, posttraumatic stress, dissociation; Trauma Symptom Checklist for Children Alternate Version [50], Child Behavior Checklist [51] (respondent: parent)
Yau (2012)	Families; ongoing prospective community study	Cohort study	NOS	20 (40)/20.2 (1.2)	Alcohol use disorder; DSM-IV (based on the father’s diagnosis)	Alcohol and substance use; Drinking and Drug History Form [42], Externalizing behavioral problems; Youth Self-Report [41] (respondent: child)

Note. NOS = not otherwise specified; approx. = approximately

The results of the assessment of methodological quality are summarized in Table 4. Due to missing information (e.g., missing descriptions of the sample recruitment strategy and diagnostic procedures used for the assessment of parental substance use disorders), the quality criteria could not be applied in most of the studies, and the quality had to be rated as *unclear*. Nevertheless, the methodological quality of five studies was rated “high” (45.5%), and the methodological quality of five other studies was rated “medium” (45.5%). The methodological quality of one study was rated “low” (9%).

**Table 4:** Methodological quality of the included studies.

First author (year)	External validity: representative sample	Measurement bias: valid & reliable measurement of		Case-control studies—selection bias: comparable study groups	Cohort studies–attrition bias:		Quality assessment
		protective factors	child’s mental health outcome		Similar dropout rate in both groups	No systematic differences between completers & non-completers	High–medium—low
Brook (2003)	(0)	(+)	(+)	(/)	(/)	(/)	High
Conners-Burrow (2012)	(0)	(+)	(0)	(/)	(0)	(0)	Medium
Edwards (2006)	(+)	(+)	(+)	(/)	(+)	(+)	High
El-Sheikh (2003)	(0)	(+)	(+)	(/)	(0)	(0)	High
Heitzeg (2008)	(0)	(+)	(0)	(+)	(/)	(/)	Medium
King (2004)	(+)	(+)	(+)	(/)	(+)	(+)	High
McCauley Ohannessian (2010)	(0)	(+)	(+)	(/)	(+)	(+)	High
Peleg-Oren (2008)	(0)	(+)	(0)	(/)	(0)	(0)	Medium
Pilowsky (2004)	(0)	(+)	(0)	(/)	(0)	(0)	Medium
Sheridan (2011)	(0)	(-)	(0)	(/)	(0)	(0)	Low
Yau (2012)	(0)	(+)	(+)	(/)	(0)	(0)	Medium

*Note*. (+) criterion fulfilled; (0) missing information/not clear; (-) criterion not met; (/) not applicable

### Protective factors

The protective factors that were identified in the 11 included studies that were associated with a positive mental health outcome in children of parents with drug or alcohol use disorders are summarized in Table 5. These factors were assigned to four categories: family, parental, child-related, or social and biological factors.

**Table 5:** Results of the systematic review.

Protective factors	Description	First author (year)	Children’s mental health outcome	
			Externalizing/ internalizing problems	Consumption/abuse of alcohol or other drugs
**Child-related factors**				
Psychological factors	Adaptive use of primary and secondary control coping	McCauley Ohannessian (2008)		+
	Approach coping strategies	Pilowsky (2004)	-	
	Ability to engage adults	Conners-Burrow (2013)	+	
Biological factors	Increased activation of the OFG and left insula	Heitzeg (2008)		+
	Blunted NAcc response	Yau (2012)		+
**Family and parental factors**				
Family factors	Family cohesion and adaptability	El-Sheikh (2003)	+	
		Peleg-Oren (2008)	-	
	Secure parent-child attachment	Brook (2003)		-
		Edwards (2006)	+	
		El-Sheikh (2003)	+	
Parental factors	Low parenting stress	Pilowsky (2004)	+	
	Accepting mother; high mother’s and low father’s controlling parenting style	Peleg-Oren (2008)	+	
		
	Parental support	King (2004)		+
	Presence of a father in addition to a mother	Conners-Burrow (2013)	+	
**Environmental factors**				
Social factors	Social support	Pilowsky (2004)	+	
		Sheridan (2011)	+	

*Note*. + = statistically significant positive association; - = No statistically significant association.

#### Child-related factors

McCauley Ohannessian and colleagues [33] examined the ability of coping clusters to predict alcohol use in children of alcoholic fathers. After a period of five years, young adults who used the coping cluster of religion, active planning and social support strategies as adolescents showed the lowest alcohol consumption [33]. This coping cluster is characterized by the adaptive use of primary control coping strategies, including planning and social support, and secondary coping strategies, including acceptance of stressful life events and religion [33]. In another study by Pilowsky and colleagues [27], approach coping strategies such as social support seeking and problem solving were used to the same extent by resilient and non-resilient children. However, resilient children were less likely to apply avoidance coping strategies such as distancing and emotion-focused coping. Furthermore, children with an ability to engage with adults to meet their needs showed lower levels of behavioral problems [25].

Two studies found a statistically significant association between specific neural activations in resilient adolescents using fMRI and an improved mental health outcome [29, 30]. Adolescents with alcoholic parents who exhibited low problem drinking behavior showed an increased activation of the orbital frontal gyrus (OFG) and left insula in response to negative affective stimuli compared to those who exhibited problem drinking behavior [30]. These brain areas are involved in monitoring appropriate behavioral responses. Heitzeg and colleagues [30] suggested that the increased activation of the OFG is associated with the increased flexibility of emotional and social behavior in resilient children of alcoholic parents.

Yau and colleagues [29] found that resilient children of alcoholic parents displayed different reward circuity. Children of alcoholics who had low alcohol use and related problems showed blunted nucleus accumbens (NAcc) incentive responsiveness compared to vulnerable controls. Furthermore, Yau and colleagues [29] reported associations among early externalizing risk, NAcc responding, and current and lifetime alcohol consumption in children of alcoholic parents. The reduced NAcc responsiveness in these children might reflect a potential resilience mechanism that reduces their risk of developing alcohol use disorders.

#### Family and parental factors

The security of the parent-child attachment was assessed in three of the included studies [24, 26, 31]. Brook and colleagues [24] examined the father-child relationship, including father warmth, adolescent identification with the father, and time spent together, as a protective factor against alcohol use in adolescents with fathers who use drugs and alcohol. Their results indicated that the father’s substance abuse was associated with warmth, identification, and time spent together, leading to lower alcohol use in the adolescent. Furthermore, the protective effect of a positive father-child bond was mediated by the youth’s deviance-prone personality, which counteracts the protective effect [24]. Edwards and colleagues [31] examined the protective effect of infant-mother attachment security in families with alcoholic fathers. Their results indicated that a secure mother-child attachment at 12 months of age had a protective effect on externalizing problems at 24 and 36 months of age and on internalizing problems at 36 months of age [31]. El-Sheikh and Buckhalt [26] assessed the protective effect of secure mother-child and father-child attachment in a community sample of families with problem drinking behavior. Their results showed that only mother-child attachment was associated with fewer externalizing problems, after controlling for demographic variables.

The results of the study by Conners-Burrow and colleagues [25] further indicated that the presence of a father in the home was statistically significantly associated with fewer behavioral problems in children of drug-using mothers. El-Sheikh and Buckhalt [26] found that higher levels of family cohesion and adaptability, including the family members’ feeling emotionally close and connected as well as the family’s ability to adapt and change in response to challenges, served as protective factors against children’s externalizing and internalizing problems. In the study by Peleg-Oren and colleagues [23], mental health outcomes did not differ between children who experienced high and low family cohesion in families with substance-abusing fathers.

Two studies highlighted the importance of parenting style for the psychological, social, and everyday home functioning and drug use disorders in children of parents with drug use disorders [23, 32]. The psychological outcome was better for children with an accepting mother. In addition, children had the best psychological outcome when the mother’s controlling parenting style was high and the father’s controlling parenting style was low [23]. Regarding social adjustment and functioning, children had better outcomes when the mother’s controlling parenting style was low and the father’s controlling parenting style was high. However, when both parents were highly controlling or not at all controlling, the children’s psychological and social adjustment were low [23]. In the study by King and Chassin [32], a supportive parenting style was found to have a protective but reactive effect on drug use disorders in children of parents with an alcohol use disorder. The protective effect was lost when the youth’s behavioral undercontrol, including impulsivity, aggressiveness, and sensation-seeking personality traits, was high. A statistically significant association was also found between lower parenting stress and resiliency in children of illicit drug-using parents [27].

#### Environmental factors

Social support was found to have a protective effect on children’s mental health in families with illicit drug-using parents [27, 28]. In the study by Pilowsky and colleagues [27], children’s self-perceived social support was statistically significantly associated with fewer mental health problems as reported by their parents. This association, however, could not be confirmed via child-reported mental health outcomes [27]. In the study by Sheridan and colleagues [28], children’s reports of support received from their grandparents were statistically significantly associated with fewer social problems and externalizing and aggressive behaviors.

## Discussion

The present review identified 11 studies that assessed 13 protective factors against mental health problems and alcohol or drug abuse in COPAD. The factors that were identified in the cross-sectional studies included the child’s ability to engage with adults, a secure parent-child attachment, family cohesion and adaptability, an accepting mother, a mother’s who is high in the controlling parenting style and a father’s who is low in the controlling parenting style, lower parenting stress and high social support for the child. Additionally, biological protective factors were identified in a case-control and a cohort study, namely increased activation in the OFG and left insula and blunted activation of the mesolimbic reward circuitry. Further, three cohort studies were included in this systematic review. In these studies, the following factors were found to be significant predictors of a better mental health outcome at a later assessment point: a secure mother-child attachment, a flexible use of coping strategies (religion, planning and search for social support) in children, and parental support. The protective effect of parental support, however, was canceled if the adolescent had a highly impulsive personality. Those findings suggest that an isolated view of protective factors without considering additional influential factors might hide essential aspects of the effectiveness of protective factors [32].

Overall, five studies assessed the protective factors of mental health in children of illicit drug-using parents. Whether these factors have predictive significance cannot be determined due to the cross-sectional design of the studies. Six studies examined protective factors in children of parents with alcohol use disorders, four of which were cohort studies. The results of these four cohort studies revealed that a secure parent-child attachment, an adaptive use of primary and secondary control coping and blunted activation of NAcc in children and parental support were protective factors against poor mental health outcomes and alcohol use disorders. A comparison of the included cross-sectional and longitudinal studies demonstrated consistent results related to a secure parent-child attachment. Comparing the results of the current review with the previous work by Park and Schepp [17] on risk and protective mental health factors in children of alcoholic parents, the following additional protective factors were identified by Park and Schepp: older age, high self-esteem, high self-regulation, flexible and positive temperaments, an optimistic attitude towards life, emotional support and extra-curricular activities. More consistent results were found with regard to appropriate parenting, a secure parent-child-attachment, religion, the presence of and interactions with meaningful family members, the presence of someone to trust, and social support.

To date, it is not easy to develop targeted and effective prevention concepts for children with parents who have an addiction based on the present knowledge. Overall, the investigation revealed protective factors that are comparable to factors that were identified for children and adolescents who are facing different kinds of developmental risks. Lee and colleagues [13] summarized and grouped protective factors relating to psychosocial resilience in youth who are facing different kinds of developmental risks in a comprehensive review. The results of the current review and the review by Park and Schepp [17] are consistent with the four main components that were identified by Lee and colleagues [13]: bonding, competence, optimism, and environment. The bonding factor includes, for example, emotional attachment and commitment to caregivers and close relationships with mature and supportive adults. The competence factor comprises good cognitive abilities, good self-regulation of emotions and impulses, positive self-perception, talents that are valued by oneself and society, and general appeal or attractiveness to others. The optimism factor includes self-efficacy, spirituality and a clear and positive identity. The environment factor comprises, for example, an organized home environment, authoritative parenting, and socioeconomic advantages [13]. Preliminary results from randomized controlled trials of interventions that target parents with drug or alcohol use disorders indicated that interventions that focus on improving parenting practices and family functioning might be effective in reducing negative consequences in affected children [18].

Limitations that need to be taken into account when interpreting the results of the current review are the considerably different sample sizes as well as the varying age of the children under examination. As almost all the studies were conducted in the United States, the generalizability of the findings to other countries is limited. It should also be noted that the assessment of children’s mental health was based on different measurements and was rated from different perspectives. In the study by Peleg-Oren and colleagues [23], for example, different results were found for the association between parenting style and children’s mental health when children’s and parents’ assessments were compared. As this review shows, there are currently only a few studies examining protective mental health factors in children of parents with drug or alcohol use disorders. Most of the studies focused on parents with alcohol, rather than illicit drug use disorders. Additionally, empirical evidence on the protective factors that were identified in this review was mainly based on the findings of one or two primarily cross-sectional studies for each factor. Therefore, the predictive significance of the identified factors is not sufficient to draw clear conclusions. Furthermore, it is difficult to compare the included studies, as it is not clear whether the analyzed children faced the same level of adverse life experiences. The risk factors for parental substance use problems might become more evident when they are associated with other risk factors. To assess the effect of protective factors on mental health outcomes, potential risk factors should be included in the assessment, as their effectiveness might be reduced at high levels of risk. Therefore, the results of the current review are preliminary and need further evaluation.

Overall, protective mental health factors in COPAD seem to be understudied. Research highlights the importance of different protective factors, with family factors, including a close and supportive parent-child relationship, as the most investigated factors in the reduction of negative mental health outcomes in affected children. Nevertheless, there is a high level of demand for future research on protective factors in children with drug- or alcohol-using parents. Further longitudinal studies that include risk and protective factors of mental health outcomes are necessary.

## Supporting information

S1 Table Prisma checklist. (DOC)

S1 Text Review protocol. (DOCX)
