# Supplementary material for: Protective mental health factors in children of parents with alcohol and drug use disorders: A systematic review
Source: PLoS One. 2017 Jun 13;12(6):e0179140. doi: 10.1371/journal.pone.0179140 (PMC5469455; doi:10.1371/journal.pone.0179140)
Supplement: S1 Text — (DOCX) [file pone.0179140.s002.docx]

**Review protocol**

**Title**

Protective factors of mental health in children of parents with alcohol and drug use disorders - a systematic review

**Review team**

Olga Wlodarczyk (OW)

Mirjam Schwarze (MS)
Julia Hellgardt (JH)

**Advisory group**

Silke Pawils
Hans-Jürgen Rumpf

Franka Metzner

**Background**

Children of parents with drug and alcohol use disorders often grow up under severe stress and are at greater risk to develop psychological and social problems. However, a substantial proportion of affected children adapt to their distressing life conditions and show a positive development of mental health. These children are described as resilient. A difference between resilient and maladapted children is the presence of protective factors. Research on resilience in children faced with parental drug and/or alcohol abuse is very important as it can inform intervention programs designed to mitigate the negative effects.

**Objective**

1. What are the protective factors in children of parents with alcohol or drug use disorder that enhance the children’s mental health outcome?

**Work plan**

- P – Population: Children of parents with drug or alcohol use disorders
  - Definition Children: all children with a mean age of ≤ 21 years that are in contact with their alcohol or drug using parent
  - Definition Parent: at least one parent that currently or in the past has had a substance-related abuse or dependence with legal and/or illicit drugs (NOT: tabbaco addiction/ behavioral addictions, e.g. pathological gambling)
  - Umgang mit komorbiden Störungen: Einschluss nur wenn klare Trennung in den Ergebnissen
- I – Intervention: no intervention study
- C – Comparator/Control
  - Children with mental health problems
  - Children without protective factors
- O – Outcome
  - Protective factors of children’s mental health outcomes
  - Children’s mental health: mental and social functioning, internalizing and externalizing problems, mental health disorders, mental health problems, drug and alcohol use disorders
  - Assessment was performed by the means of a standardized instrument that allows a valid and reliable assessment
- Studiendesign
  - Observational studies
    - retrospective and prospective cohort studies
    - cross-sectional studies
    - case-control studies
    - no intervention studies

**Search strategy**

- Electronic databases: PsychInfo, PubMed, ISI Web of Science, CINHAL
- Period: January 2000 – January 2017

**Search terms**

- *Protective Factor* (protective factor* OR resilien* OR coping) AND
- *Children* (child* OR infant* OR offspring* OR adolescent* OR son* OR daughter*) AND
- *Parents* (parent* OR mother* OR father* OR maternal OR paternal) AND
- *Substance* (substance* OR drug* OR alcohol* OR opioid* OR amphetamine* OR cannabis* OR sedative* OR tranquilizer* OR cocaine*OR hallucinogen* OR heroin* OR hypnotic*OR marijuana OR psychedelic* OR phencyclidine OR narcotic OR illicit drug* abus* OR misuse OR dependence OR substance disorder OR addiction)

**Study selection**

**Table 1. Inclusion criteria (IC).**

| Preselection | | |
| --- | --- | --- |
| IC 0 | (1) the study is concerned with children of addicted parents and (2) the children, not the parents, are central in the study. | |
| Outcome: Association between at least one protective factor and child’s mental health | | |
| IC 1 | Protective factor: at least one factor is examined that protects or strengthens … | |
| IC 2 | … the child’s mental health (mental and social functioning) | |
| Population: children of families in which at least one parent has an alcohol or drug use disorder (NOT: specially strained samples, e.g. war, flight, physical impairment/disease, mental disability, child abuse, drug use during pregnancy) | | |
| IC 3 | Children: all children and adolescents with a mean age of ≤ 21 years that are in contact with | |
| IC 4 | Parents: at least one parent that currently or in the past has had a substance-related abuse or dependence with legal and/or illicit drugs (NOT: tabbaco addiction/ behavioral addictions, e.g. pathological gambling) | |
| IC 5 | Is the study population of the control group (case-control-studies) or the initial population (cohort studies) made up with children with lower levels of mental health and … | |
| IC 6 | … children with lower levels of examined protective factor. | |
| Publication | | |
| IC 7 | Is the study original, empirical research published in a peer reviewed journal (NOT: dissertations, congress contribution) | |
| Study design | | |
| IC 8 | Is the design of the study one of the following: | |
|  | (retrospective or prospective) cohort study? OR: | |
|  | Case-control study? OR: | |
|  | Cross-sectional study? | |
|  | NOT: intervention studies | |
| Diagnostics | | |
| IC 9 | | Parents: Was formal diagnostics for a substance abuse disorder or addiction performed in accordance with DSM-III, DSM-IV, DSM-IV-TR, ICD-9, ICD-10, RDC OR by the means of an instrument that allows a valid and reliable diagnosis OR by the applicaion of an urine test OR based on the long term use of a substance with a high addictiveness (e.g. heroin, methamphetamin) OR was the sample recruited from bigger longitudinal or interventional studies evalueting the effectiveness of addiction treatments? (NOT: Parental diagnosis based only on children’s perspective) |
| IC 10 | | Children: Was the assessment of the child’s mental health performed by the means of a standardized instrument that allows a valid and reliable assessment? |

**Table 2. Assessment of the methodological quality of included studies.**

| External validity | | YES | NO | UNCLEAR | NOT APPLICABLE |
| --- | --- | --- | --- | --- | --- |
| 1. | Are the study participants representative of the population under study? | (+) | (-) | (0) | (/) |
| Measurement bias | | | | | |
|  | Were valid and reliable measurement used to assess: | | | | |
| 2. | … protective factors? | (+) | (-) | (0) | (/) |
| 3. | … parental drug abuse disorder or addiction? | (+) | (-) | (0) | (/) |
| Attrition bias (cohort studies) | |  |  |  |  |
| 4. | Were the drop-out rates in both groups acceptable (≤ 20%)? | (+) | (-) | (0) | (/) |
| Selection bias (case-control-studies) | | | | | |
| 5. | Were participants in both groups comparable with respect to possibles confounders? | (+) | (-) | (0) | (/) |

**Table 4. Methodological quality of the included studies.**

| **First author (year)** | **External validity:**  **representative sample** | **Measurement bias: valid & reliable measurement of** | | **Case-control-studies - selection bias: comparable study groups** | **Cohort studies – attrition bias:** | | **Quality assessment** |
| --- | --- | --- | --- | --- | --- | --- | --- |
|  |  | **protective factors** | **child mental health outcome** |  | **Similar drop-out rates in both groups** | **No systematic differences between completers & drop-outs** | **High – medium - low** |
| Brook (2003) | (0) | (+) | (+) | (/) | (/) | (/) | High |
| Conners-Burrow (2012) | (0) | (+) | (0) | (/) | (0) | (0) | Medium |
| Edwards (2006) | (+) | (+) | (+) | (/) | (+) | (+) | High |
| El-Sheikh (2003) | (0) | (+) | (+) | (/) | (0) | (0) | High |
| Heitzeg (2008) | (0) | (+) | (0) | (+) | (/) | (/) | Medium |
| King (2004) | (+) | (+) | (+) | (/) | (+) | (+) | High |
| McCauley Ohannessian (2010) | (0) | (+) | (+) | (/) | (+) | (+) | High |
| Peleg-Oren (2008) | (0) | (+) | (0) | (/) | (0) | (0) | Medium |
| Pilowsky (2004) | (0) | (+) | (0) | (/) | (0) | (0) | Medium |
| Sheridan (2011) | (0) | (-) | (0) | (/) | (0) | (0) | Low |
| Yau (2012) | (0) | (+) | (+) | (/) | (0) | (0) | Medium |

*Note*. (+) criterion fulfilled; (0) missing information/not clear; (-) criterion not met; (/) not applicable


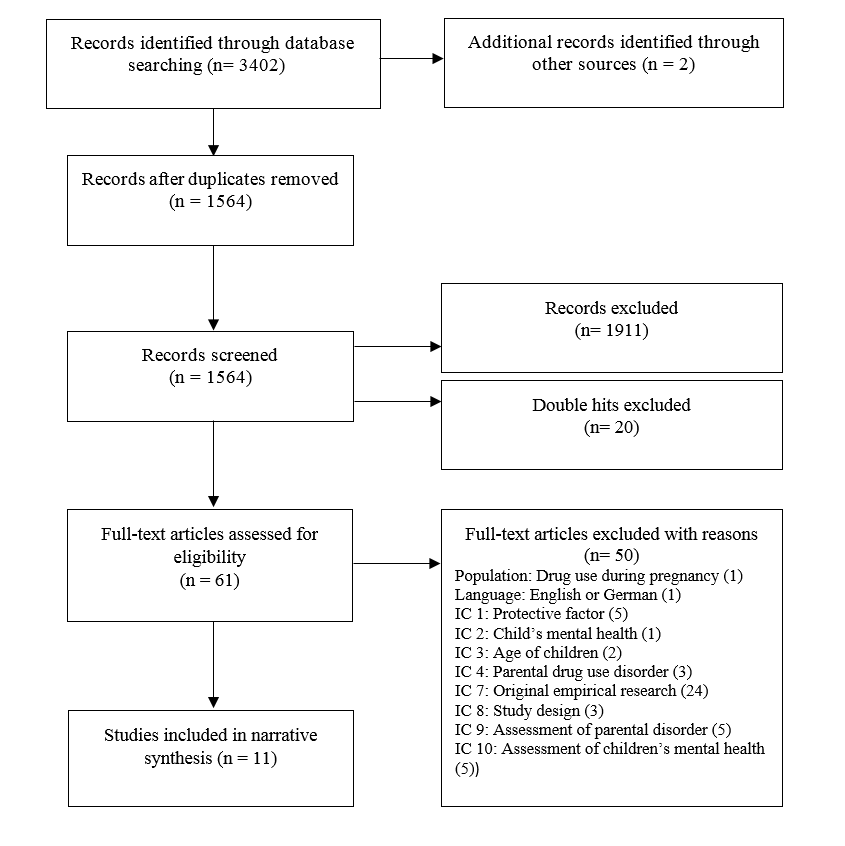


**Fig. 1. Flowchart of information through the different phases of the systematic review.**
